# Vitamin D deficiency and surgical cellulose mimicking parathyroid adenoma after initial parathyroidectomy

**DOI:** 10.55730/1300-0144.6017

**Published:** 2024-12-23

**Authors:** Mustafa ŞAHİN, Volkan GENÇ, Koray CEYHAN, Demet ÇORAPÇIOĞLU

**Affiliations:** 1 Department of Endocrinology and Metabolism, Faculty of Medicine, Ankara University, Ankara, Turkiye; 2 Department of General Surgery, Faculty of Medicine, Ankara University, Ankara, Turkiye; 3 Department of Pathology, Faculty of Medicine, Ankara University, Ankara, Turkiye

**Keywords:** Surgical cellulose, parathyroid adenoma, surgery

## Abstract

**Background/aim:**

Primary hyperparathyroidism is a condition characterized by an excessive production of parathyroid hormone primarily resulting from the presence of an adenoma or hyperplasia of the parathyroid glands.

**Materials and methods:**

A 45-year-old woman who had previously experienced high levels of calcium in her blood and abnormally elevated parathyroid hormone levels underwent surgical removal of the left parathyroid gland. Following surgery, she exhibited high levels of parathyroid hormone along with normalized calcium levels and persistent fatigue. Ultrasound imaging showed a hypoechoic mass on the left side, which was initially considered to be a recurrent parathyroid tumor.

**Results:**

A comprehensive assessment, including neck CT scans and fine needle aspiration cytology, revealed that the mass was actually residual surgical cellulose, making diagnosis more challenging due to vitamin D deficiency.

**Conclusion:**

Following parathyroid surgery, surgical cellulose may mimic a parathyroid adenoma.

## 1. Introduction

Primary hyperparathyroidism (PHPT) is a disorder characterized by the overproduction of the parathyroid hormone (PTH). It is commonly caused by adenoma or hyperplasia of the parathyroid glands [1]. High calcium levels and elevated or inappropriately normal levels of parathyroid hormone are characteristics of hyperparathyroidism [1]. Parathyroidectomy, the surgical removal of the parathyroid adenoma, is the standard treatment for patients with symptomatic PHPT or if other surgical indications in asymptomatic patients exist [2]. While parathyroidectomy is highly successful in resolving the condition, recurrent or persistent hyperparathyroidism can occur in a subset of patients, presenting diagnostic challenges [2]. We generally measure serum calcium and PTH levels and perform parathyroid sonography to exclude recurrent and persistent disease. Recurrent or persistent hyperparathyroidism may be observed with hypercalcemia and elevated or inappropriately normal PTH levels. Parathyroid sonography is also considered in the follow-up of patients after parathyroidectomy. Parathyroid adenoma typically appears as well-defined, hypoechoic, or isoechoic nodules on ultrasound with typical polar vascularization in Doppler sonography [2].

Oxidized cellulose (OC) is a biodegradable polymer used in bleeding hemostasis in thyroid and parathyroid surgeries [3]. OC may mimic recurrent disease in thyroid cancer patients who have undergone surgery [3]. The ultrasound characteristics of OC may vary depending on the type of OC product, its location in the body, and duration after surgery [4]. According to OC type, it may take a long time to degrade. Some case studies have reported that OC is mistakenly diagnosed as recurrent thyroid cancer in patients after thyroid surgery [4].

This is the first documented case of a misdiagnosis of OC as a parathyroid adenoma in a patient with vitamin D deficiency following parathyroid surgery. This case highlights the challenges of managing recurring high calcium levels in patients who have previously undergone parathyroid surgery and later developed vitamin D deficiency and a surgical cellulose mimicking a parathyroid adenoma

## 2. Case description

**Figure 1:**
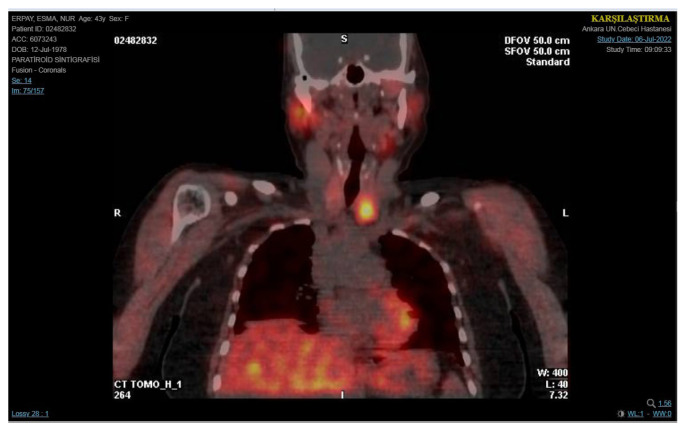
Four-dimensional tomography and the sestamibi scan of focal uptake in the left neck region, suggesting the presence of hyperfunctioning parathyroid tissue.

A 45-year-old female presented with high calcium levels and increased parathyroid hormone (PTH) concentrations. Neck ultrasonography revealed a hypoechoic solid 13 × 8 × 5 mm hypoechoic lesion with polar vascularization in the left inferior region and a 7 × 4 × 2 mm thyroid nodule in the right thyroid lobe. Four-dimensional tomography (4D-CT) and a sestamibi scan revealed a focal increase in activity in the left neck area, indicating the presence of hyperfunctioning parathyroid tissue (Figure 1).

The patient’s bone mineral density measurements were osteopenic in the distal radius. Her calcium level and urinary calcium levels are shown in Table. She underwent minimal invasive left parathyroidectomy on 20.07.2022. Pathology revealed an atypical adenoma in the left parathyroid.

The pathology findings suggested a parathyroid adenoma with atypical features. The pathological findings were the following: acinar organized lesion consisting of parathyroid cells with an encapsulated appearance. There was an oncocytic change in some of the cell groups, occasional infiltration in the capsule, and suspicion of lymphovascular invasion. Chromogranin A and PTH were positive, synaptophysin was negative, and the Ki-67 proliferation index was <1%. There were two mitoses per 2 mm^2^. Galectin expression was positive in three periphery capsules; RB and parafibromin were preserved; CD61 was negative, but CD34 was positive in the capsule.

The initial surgery aimed to remove a parathyroid adenoma, and surgical cellulose was used as a hemostatic agent during the procedure.

**Figure 2:**
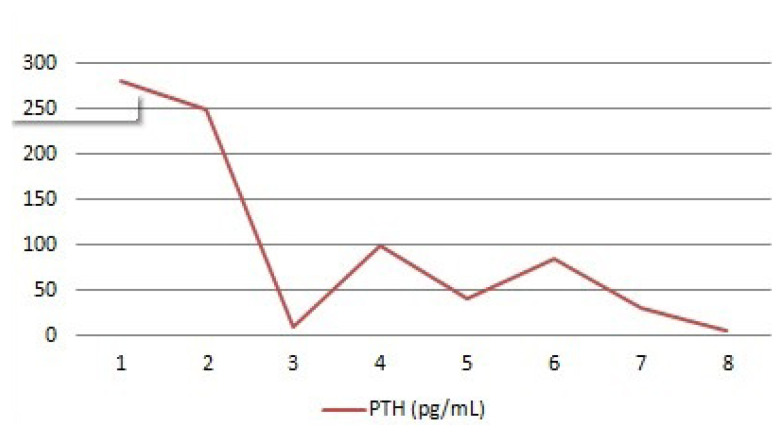
PTH levels during the follow-up period.

Three months after parathyroidectomy surgery, the patient returned to the Endocrinology Department with increased parathyroid hormone (PTH) levels, but her calcium levels remained normal. Further laboratory tests showed persistently high levels of PTH, and the patient’s calcium levels continued to be at the upper limit of the normal range (Table and Figure 2). Figure 2 shows PTH levels during the follow-up period. Additionally, the patient had been experiencing chronic fatigue and musculoskeletal pain, prompting further evaluation.

**Figure 3:**
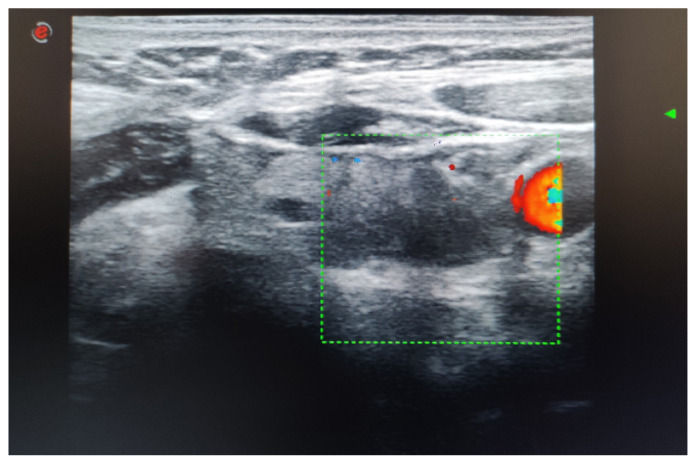
Ultrasonography of a well-defined ovoid lesion in the surgical bed after 3 months of parathyroid surgery.

**Figure 4:**
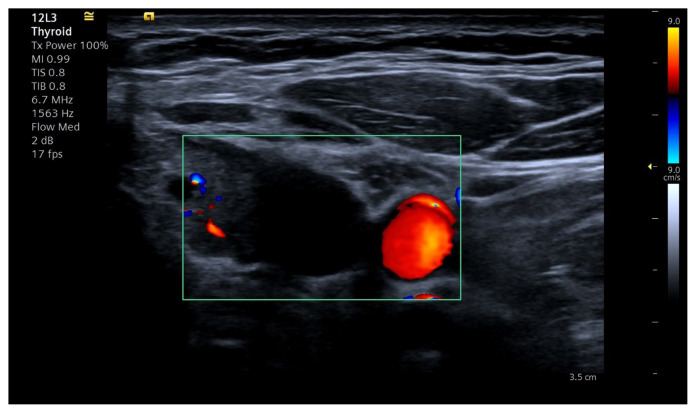
Ultrasonography of a well-defined ovoid lesion in the surgical bed after 12 months of parathyroid surgery.

Combining postsurgical neck ultrasonography results with PTH levels offers essential data for ongoing assessment. The ultrasonography examination revealed a hypoechoic nodule in the right lobe, measuring 5 × 9 × 10 mm, with a regular border and no halo present, corresponding to a TIRADS 4. Additionally, two nodules smaller than 1 cm were found in the left lobe, each containing macrocalcifications corresponding to a TIRADS 3. A hypoechoic lesion measuring 8 × 11 × 18 mm was found in the left inferior area, external to the thyroid gland, and lacking vascularization (Figures 3 and 4).

## 3. Diagnostic challenge

The main difficulty in diagnosing this case was the coexistence of vitamin D deficiency and the ultrasonographic appearance of a suspicious hypoechoic lesion on the left side of the neck, mimicking parathyroid adenoma. Moreover, the patient’s vitamin D levels were significantly below the normal range.

A differential diagnosis between parathyroid adenoma and the OC product might necessitate a thorough examination of clinical history, surgical history, physical examination, laboratory tests, as well as potentially scintigraphy and fine needle aspiration. Accurate diagnosis is crucial for appropriate management. When assessing the presence of OC residue, it is essential to consider the patient’s surgical history. As OC is an inorganic tissue, color Doppler imaging will not show internal vascularization.

## 4. Clinical management

**Figure 5:**
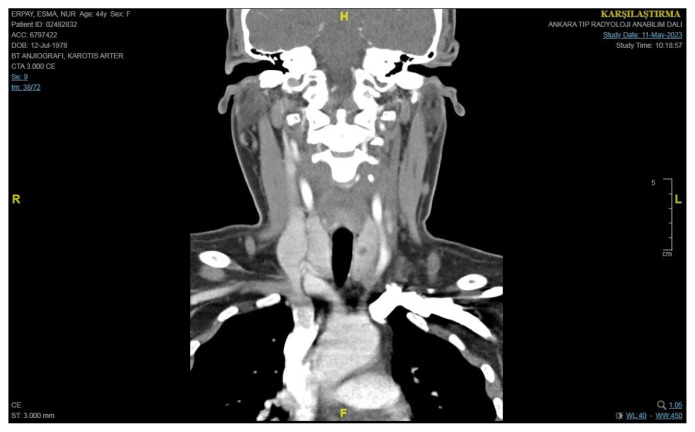
CT scan of a hypodense mass (diameter 19 × 10 × 32 mm) of cellulose.

**Figure 6:**
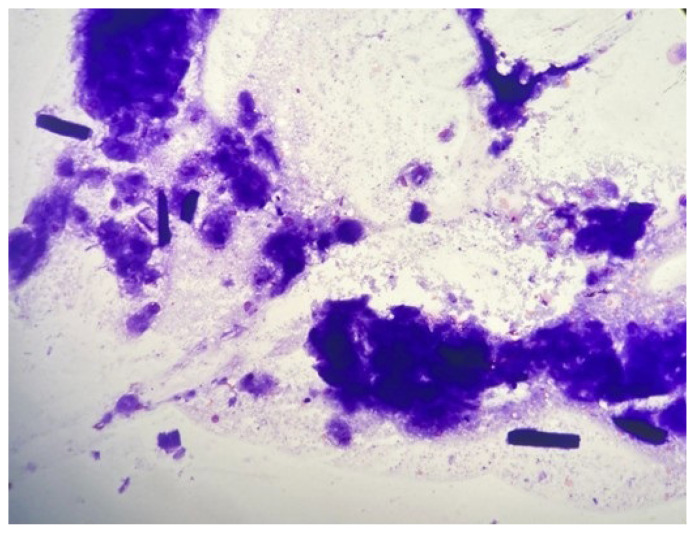
Fine needle aspiration cytology of the postoperative pseudo parathyroid adenoma lesion (oxidized cellulose hemostatic agents) and oxidized cellulose elongated fragments.

Given the diagnostic dilemma in our study’s case, a comprehensive evaluation was initiated. Further imaging, with a CT scan of the neck, was performed. The CT scan demonstrated the presence of a hypoechoic structure consistent with surgical cellulose from a previous procedure (Figure 5). The CT scan showed no suspicious parathyroid lesion, but a left hypodense mass was visible (diameter: 19 × 10 × 32 mm) without postcontrast enhancement. TSH was 2.75 (0.27–4.2 IU/mL), thyroglobulin washout was 0.13, the serum Tg level was 0.53 ng/mL, and PTH washout was 5 pg/mL from the left hypoechoic lesion. There was a consideration of the combination of vitamin D deficiency and persistent hyperparathyroidism. Fine needle aspiration cytology (FNAC) of the left hypoechoic mass was performed, and the cytological examination showed only amorphous material. FNAC showed pathological cellulose structures (Figure 6).

## 5. Outcome

Imaging studies showed that a parathyroid adenoma was imitating local inflammation caused by residual surgical cellulose. Therefore, the patient received vitamin D supplementation to correct the deficiency. Following vitamin D supplementation, the patient’s PTH levels normalized. In addition, the patient’s fatigue and musculoskeletal pain symptoms considerably improved.

## 6. Conclusion

This case underscores the importance of considering the impact of vitamin D deficiency on parathyroid function and recurrent PTH increase, even in patients with a history of parathyroidectomy. The use of surgical cellulose also raises the possibility that it could be mistaken for a parathyroid adenoma on imaging tests, thereby complicating the diagnostic process. A multidisciplinary approach, including comprehensive imaging and FNAC when necessary, is crucial for accurate diagnosis and effective management.

## Figures and Tables

**Table:** Laboratory results of the patient during the follow-up period.

	At presentation 20.06.2022	After parathyroidectomy 21.07.2022	3 months after parathyroidectomy 10.10.2022	Patient’s result after D3 therapy 16.09.2023	Reference range
**Calcium (mg/dL)**	11.4	9.8	9.8	9.4	8.6–10.2
**Albumin (g/L)**	42.4	36.6	36.6	42.3	35–52
**Phosphate (mg/dL)**	1.97	2.71	2.62	3.5	2.5–4.5
**Magnesium (mg/dL)**	1.7	1.7	1.81	2.03	1.6–2.6
**25-hydroxy vitamin D3 (mg/L)**	17.4	12.66	10.31	22	
**Parathyroid hormone (pg/mL)**	280	9	99	29	15–65
**Alkaline phosphatase (U/L)**	185			81	35–105
**Urinary calcium level (mg/24 h)**	276			102	100–300
**Urinary phosphate level**	360			482.4	400–1300
**Glomerular filtration rate mL/min/1.73 m** **^2^**	116			102	
