# Erratum to “Vitamin D deficiency and surgical cellulose mimicking parathyroid adenoma after initial parathyroidectomy” [Turkish Journal of Medical Sciences 55 3 696 701]

**DOI:** 10.55730/1300-0144.6058

**Published:** 2025-09-01

**Authors:** Mustafa ŞAHİN, Volkan GENÇ, Koray CEYHAN, Demet ÇORAPÇIOĞLU

**Affiliations:** 1 Department of Endocrinology and Metabolism, Faculty of Medicine, Ankara University, Ankara, Turkiye; 2 Department of General Surgery, Faculty of Medicine, Ankara University, Ankara, Turkiye; 3 Department of Pathology, Faculty of Medicine, Ankara University, Ankara, Turkiye

We have identified that the article type of our publication entitled “Vitamin D deficiency and surgical cellulose mimicking parathyroid adenoma after initial parathyroidectomy” was mistakenly entered incorrectly during the submission process.

This error occurred due to an oversight at the time of submission.

The correct article type should be Case Report instead of the previously indicated type.

This correction does not affect the scientific content, results, or conclusions of the article.

We apologize for any inconvenience this may have caused to our readers and the indexing services.

